# Funding has no effect on clinical outcomes of total joint arthroplasty emerging technologies: a systematic review of bibliometrics and conflicts of interest

**DOI:** 10.1186/s42836-022-00146-3

**Published:** 2022-11-01

**Authors:** Garrhett G. Via, David A. Brueggeman, Joseph G. Lyons, Isabelle C. Ely, Andrew W. Froehle, Anil B. Krishnamurthy

**Affiliations:** Department of Orthopedic Surgery, Wright State University, 30 E. Apple St., Ste 2200, Dayton, OH 45409 USA

**Keywords:** Conflict of interest, Funding, Total joint arthroplasty, Robotic-assisted, Computer-navigated, Patient-specific implant

## Abstract

**Background:**

The use of new total joint arthroplasty technologies, including patient-specific implants/instrumentation (PSI), computer-assisted (CA), and robotic-assisted (RA) techniques, is increasing. There is an ongoing debate regarding the value provided and potential concerns about conflicts of interest (COI).

**Methods:**

PRISMA guidelines were followed. PubMed, MEDLINE, and Web of Science databases were searched for total hip and knee arthroplasties, unicompartmental knee arthroplasties (UKA), PSI, CA, and RA. Bibliometric data, financial COI, clinical/functional scores, and patient-reported outcomes were assessed.

**Results:**

Eighty-seven studies were evaluated, with 35 (40.2%) including at least one author reporting COI, and 13 (14.9%) disclosing industry funding. COI and industry funding had no significant effects on outcomes (*P* = 0.682, *P* = 0.447), and there were no significant effects of conflicts or funding on level of evidence (*P* = 0.508, *P* = 0.826). Studies in which author(s) disclosed COI had significantly higher relative citation ratio (RCR) and impact factor (IF) than those without (*P* < 0.001, *P* = 0.032). Subanalysis demonstrated RA and PSI studies were more likely to report COI or industry funding (*P* = *0.045*). RA (OR = 6.31, 95% CI: 1.61–24.68) and UKA (OR = 9.14, 95% CI: 1.43–58.53) had higher odds of reporting favorable outcomes than PSI.

**Conclusions:**

Author COIs (about 40%) may be lower than previously reported in orthopedic technologies/techniques reviews. Studies utilizing RA and PSI were more likely to report COI, while RA and UKA studies were more likely to report favorable outcomes than PSI. No statistically significant association between the presence of COIs and/or industry funding and the frequency of favorable outcomes or study level of evidence was found.

**Level of evidence:**

Level V Systematic Review

**Supplementary Information:**

The online version contains supplementary material available at 10.1186/s42836-022-00146-3.

## Introduction

Although not uniform in their adoption by orthopedic surgeons, utilization of emerging total joint arthroplasty (TJA) technologies is increasing [1]. While the use of new technologies, including patient-specific implants/instrumentation (PSI), computer-assisted (CA), and robotic-assisted (RA) techniques, is increasing, there is an ongoing debate regarding their value. Critiques of using PSI in TJA include inconsistent improvement of functional outcomes, increased patient preoperative waiting times, and additional costs [2–4]. Compared to PSI and conventional methods, CA navigation and RA TJA methods have demonstrated improved accuracy and precision of component positioning [2, 5–10]. Despite reliable improvements in radiological outcomes, most research to date has found no significant difference in functional outcomes between CA, RA, *vs*. conventional methods [5–7, 10–12]. However, select studies support the potential for outcome and implant survivorship advantages with these methods [9, 13]. More high-quality studies are needed to assess whether emerging technologies improve clinical/functional outcomes and implant survivorship after TJA compared to conventional techniques.

Considering the rapid expansion and emergence of new technologies, the issue of funding new research is paramount. Development and implementation of clinical trials for new techniques are costly, and may be inherently associated with the presence of financial conflicts of interest (COI) [14]. Acknowledging the importance of recognizing COIs in biomedical research, the Centers for Medicare and Medicaid Services (CMS) Open Payments program was enacted within the Physician Payment Sunshine Act of 2007. This database reveals prevalent and significant financial relationships between industry and orthopedic surgeons [15, 16]. Albeit important for innovation and advancement, these relationships remain concerning with regard to their influence on study quality and design, reporting of results, and publication of positive findings [17].

Financial COI, undeclared payments, and studies with discrepancies in reported and actual COI appear to be associated with reporting favorable results with RA [18, 19]. Manuscripts on RA in unilateral knee arthroplasty (UKA) manuscripts were more likely to have authors with COIs or industry funding, and were more likely published in less prestigious journals [20]. Similarly, in RA TJA, 91% of relevant manuscripts had an author financial COI, and nearly all studies reporting favorable outcomes had an author with a COI [21]. Emerging TJA technologies have the potential to benefit surgeons and patients alike, but it is critical to ensure that informed decision-making and evidence-based medicine prevail. The demonstrated prevalence of COIs in the field of orthopedic surgery raises the concern that COI may influence study outcomes.

To address this concern, we conducted a systematic review to assess bibliometric data, financial COIs, and overall outcomes of manuscripts involving emerging TJA technologies. Previous studies have investigated the prevalence and impact of COIs on robotic TJA research [20, 21]. We sought to expand upon prior work by exploring the influence of COIs on not just robotic TJA research, but on all new and innovative TJA technologies. Furthermore, we sought to analyze only those studies which reported non-radiographic functional and/or patient-reported outcomes. The intent behind these more stringent inclusion criteria was to explore what influence, if any, financial COI may have on the results and conclusions of studies reporting only those outcome measures which may be most clinically meaningful to patients and physicians. This evidence may better inform shared decision-making in the care of patients when considering the use of new techniques/technologies. Our primary hypotheses were that manuscripts favorably detailing an emerging TJA technology were more likely to have a COI, that funded/conflicted studies are of higher quality and/or are published in higher quality journals, and that there are correlations between study outcomes, level of evidence, and publication quality.

This systematic review was conducted in accordance with the Preferred Reporting Items for Systematic Reviews and Meta-Analyses (PRISMA) statement, a guideline which was developed and recently updated to facilitate transparent and complete reporting of systematic review methodology and terminology [22]. The PRISMA statement recommendations have been widely endorsed and adopted by the biomedical research community and represent the gold standard approach to reporting systematic reviews [23].

## Methods

### Literature search and inclusion criteria

Following PRISMA guidelines, we performed a systematic review of emerging technologies in TJA published from January 2011 to April 2021 within the PubMed, MEDLINE, and Web of Science databases. Articles from this time period were chosen to coincide with the passage of the Physician Payments Sunshine Act to ensure that the most complete information regarding potential COI would be available. Topics of interest included total hip and knee arthroplasties, unicompartmental knee arthroplasties, robotics, computer-assisted, and patient-specific implants.

The final search strategy for publications utilized in the databases was “((((Total joint arthroplasty OR TJA OR Total knee arthroplasty OR TKA OR Total hip arthroplasty OR THA OR Unicompartmental arthroplasty OR UKA) AND (Robot-assisted OR Robotic-assisted OR Computer navigated OR Computer assisted OR Customized implant OR Technology assisted OR Patient specific implant OR patient specific instrument* OR PSI OR Mako OR Mazor OR Rosa OR Navio OR Excelsius OR Conformis) NOT (Shoulder OR Spine OR Pedicle OR Elbow OR Ankle OR Revision OR Biomechanic* OR Cadaver OR *in vitro* OR Animal OR *in vivo* OR Commission OR TMJ OR Hand OR Wrist OR Arthroscop* OR Laparoscop* OR Labrum OR Obstet* OR Gynec* OR Cardiol* OR Heart OR Disease OR Tumor OR Oncology OR Radiation OR Radiology OR Sacroiliac OR Biochem* OR Botan* OR Food OR ATLAS)) NOT (review[Publication Type])) NOT (systematic review[Publication Type])) NOT (meta analysis[Publication Type]) NOT (editorial[Publication Type]).” This was completed in March 2021.

Screening of articles was conducted in 3 stages using the web-based version of Rayyan Intelligent Systematic Review (Rayyan Systems Inc., Qatar Computing Research Institute, Doha, Qatar). The stages of review included screening articles based on title, abstract, and then full-text respectively. Screening was conducted by 3 independent reviewers with a requirement of 2 out 3 selection for inclusion in the study during each stage. Only articles published in English and with full-text availability were eligible for inclusion.

Manuscripts were included only if they reported functional and/or patient-reported outcomes utilizing a validated scoring tool and only if they included a statistical comparison between conventional instrumentation and the emerging technology of interest. These were included because we believed that patient functional and reported outcomes represented the most clinically meaningful domains considered by both patients and physicians to inform shared decision-making. Studies reporting only radiographic (*e*.*g*., accuracy of component positioning) and/or non-functional clinical outcomes (*e*.*g*., intraoperative blood loss, surgical time) were excluded. These outcomes were excluded as their clinical relevance has not been fully elucidated and because we believed that they would not ordinarily be weighed heavily when informing patient decision-making [24–27].

**Fig. 1:**
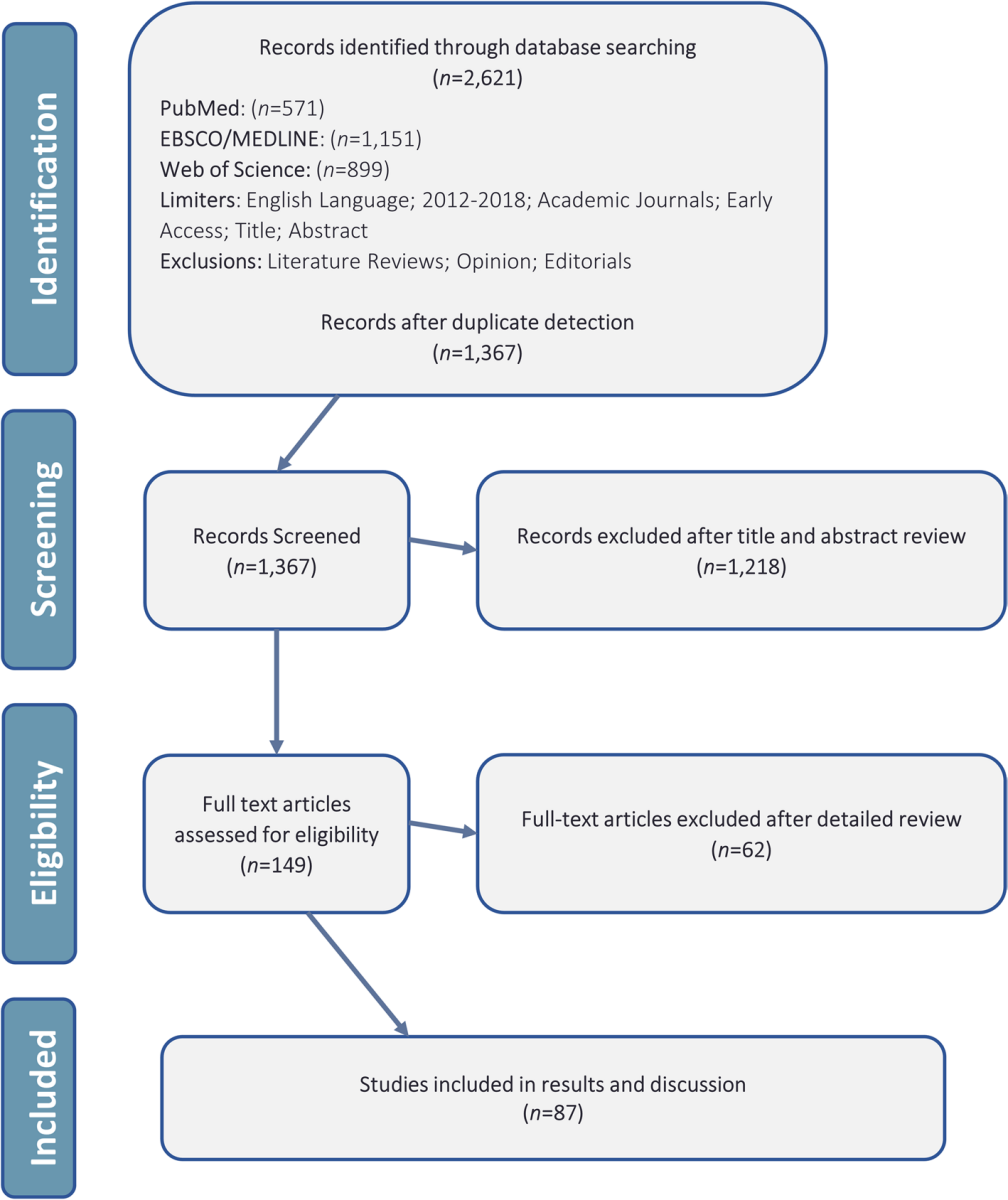
PRISMA flow diagram for emerging TJA technologies

Biomechanical and cadaveric studies were also excluded from this analysis. Commentaries, editorials, case reports, systematic reviews, meta-analyses, and clinical studies with less than 20 patients per treatment arm were excluded from this study. Articles without a disclosures section or COI statement were also excluded. The literature search methods and inclusion/exclusion criteria were developed in accordance with similar studies successfully published in the field [20, 21, 28]. Figure 1 depicts the PRISMA flow diagram for the screening of manuscripts in the present study.

### Data collection and outcome measures

Final data for analysis were collected upon full-text review of the selected manuscripts. The publication year, author names, open access status, industry funding status and industry of interest (if applicable), the relevant study years from data collection until publication (for industry conflict purposes), impact factor (IF; InCites Journal Citations Report), and relative citation ratio (RCR; NIH iCite) were documented [29]. Level of evidence was collected and determined based on the guidelines established by the Journal of Bone and Joint Surgery.

Author COIs for each study were identified using two methods. The disclosure sections for the manuscripts were reviewed for self-reported COIs and/or industry funding. Additionally, all (non-international, US-based) authors were searched in the online CMS Open Payments reporting database. Each author was reviewed for officially disclosed monetary payments, stock ownership, and/or research funding. Reported COIs were categorized as directly related to the study if there were associations with the industry/manufacturer that produced the technology/implants referenced in the study. Additionally, the amounts of monetary payments/funding during the respective study years were collected for further analysis.

Each manuscript was identified as having a favorable, equivocal, or unfavorable outcome. Studies with favorable outcomes included those demonstrating statistically significant (*P* < 0.05) superiority of the emerging TJA technology/implant over a control. Studies with equivocal results included those with no statistical differences or those with inconclusive results. Studies with unfavorable outcomes included those demonstrating inferiority (*P* < 0.05) or absence of clinical benefits of the emerging TJA technology/implant compared to a control. Metrics for review and outcome characteristics were adapted from similarly-oriented research [20, 21, 28].

### Statistical analysis

All statistical analyses were performed by using SAS 9.4 (SAS Institute, Cary, NC), and significance was set at *α* = 0.05. For categorical (study outcome) and ordinal (level of evidence) variables, frequencies were compared between groups using Pearson Chi-square tests. For continuous variables (RCR, IF, total payments), groups were compared using non-parametric Wilcoxon-Mann-Whitney tests, since data were not normally distributed.

## Results

**Fig. 2:**
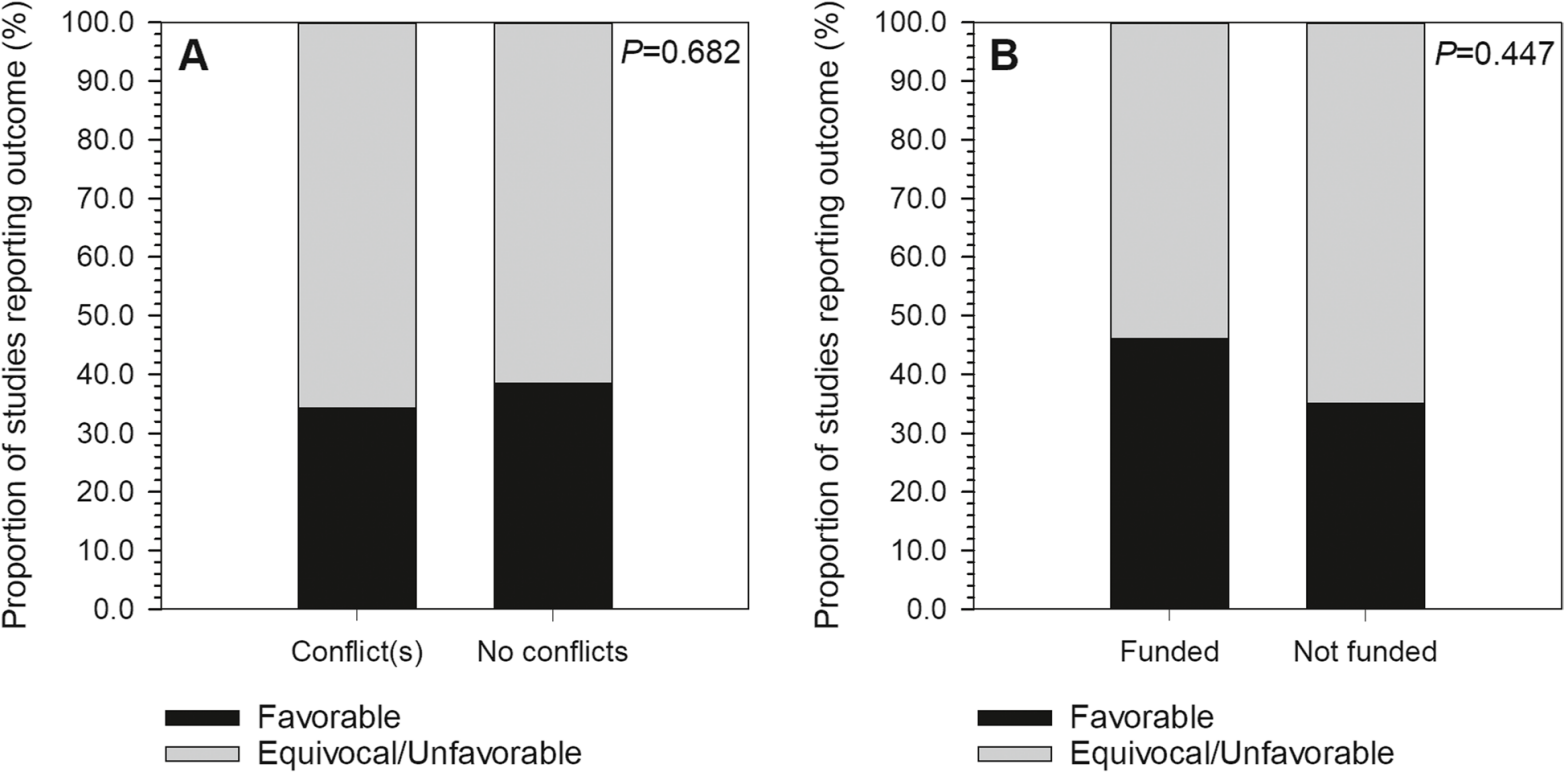
Proportions of studies reporting favorable outcomes (black bar segments) or equivocal/unfavorable outcomes (gray bar segments). Studies were compared for (**A**) any reported conflicts of interest *vs*. no reported conflicts of interest, and (**B**) any reported industry funding *vs*. no reported industry funding. Neither comparison found significant between-groups differences

**Fig. 3:**
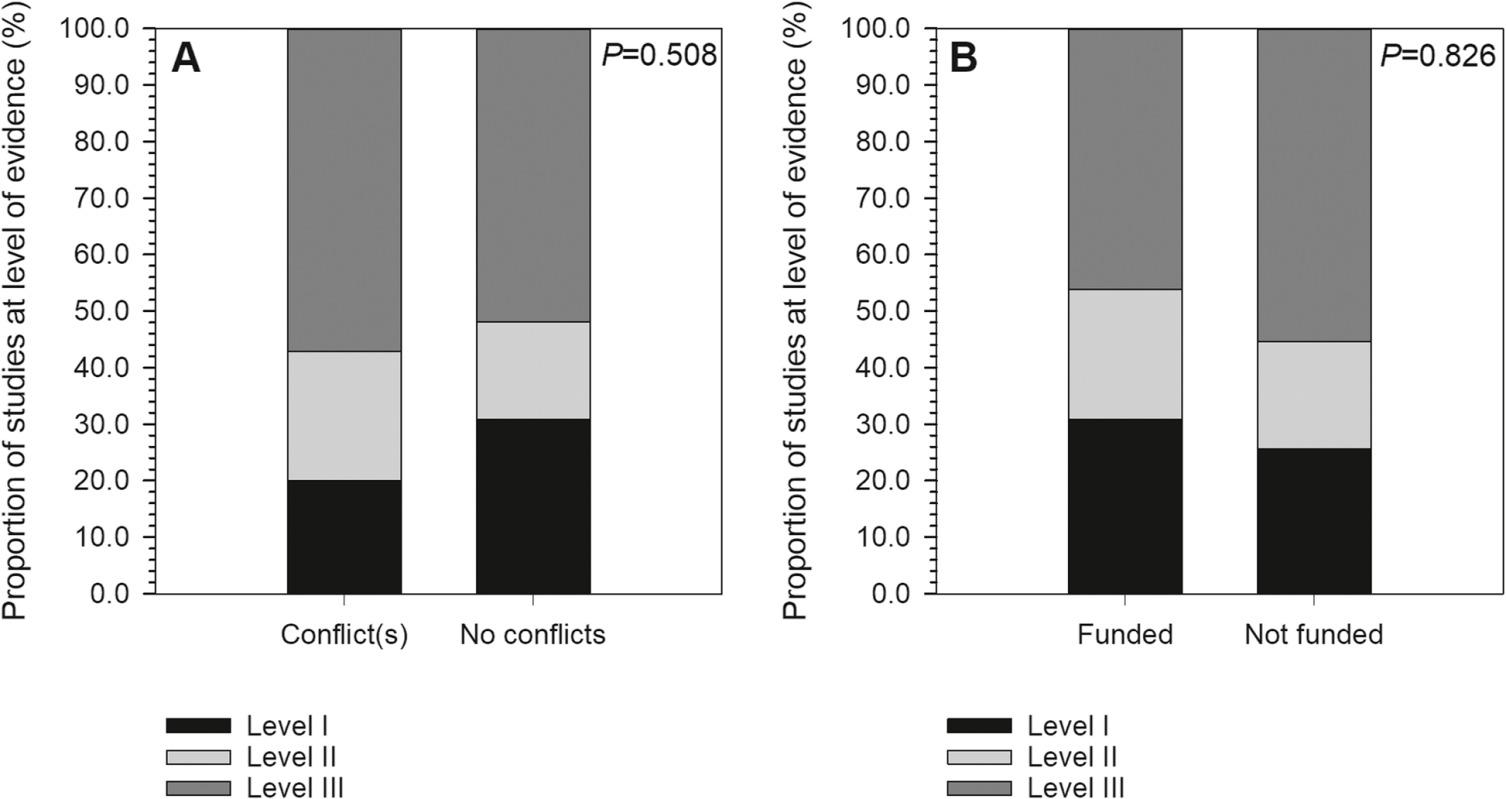
Proportions of studies reporting Level I evidence (black bar segments), Level II evidence (light gray bar segments) or Level III evidence (dark gray bar segments). Studies were compared for (**A**) any reported conflicts of interest *vs*. no reported conflicts of interest, and (**B**) any reported industry funding *vs*. no reported industry funding. Neither comparison found significant between-groups differences

**Fig. 4:**
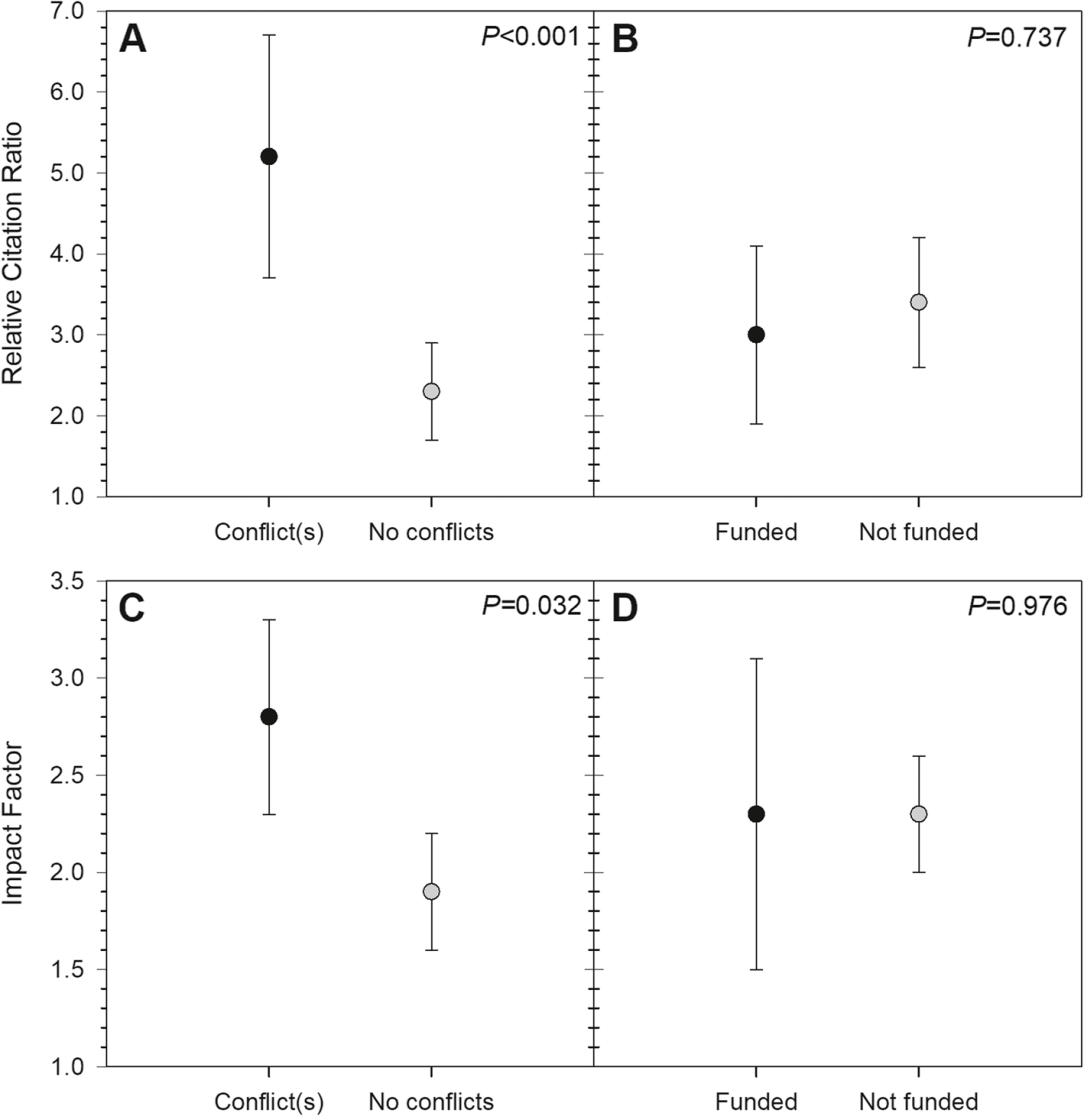
Means (circles) and their 95% confidence intervals (error bars) for Relative Citation Ratio (RCR) and Impact Factor (IF). Studies were compared for any reported conflicts of interest *vs*. no reported conflicts of interest (**A**: RCR; **C**: IF), and any reported industry funding *vs*. no reported industry funding (**B**: RCR; **D**: IF). Studies reporting conflicts of interest tended to have significantly higher RCR and IF than those reporting no conflicts. Funded and unfunded studies did not differ significantly for RCR or IF

**Fig. 5:**
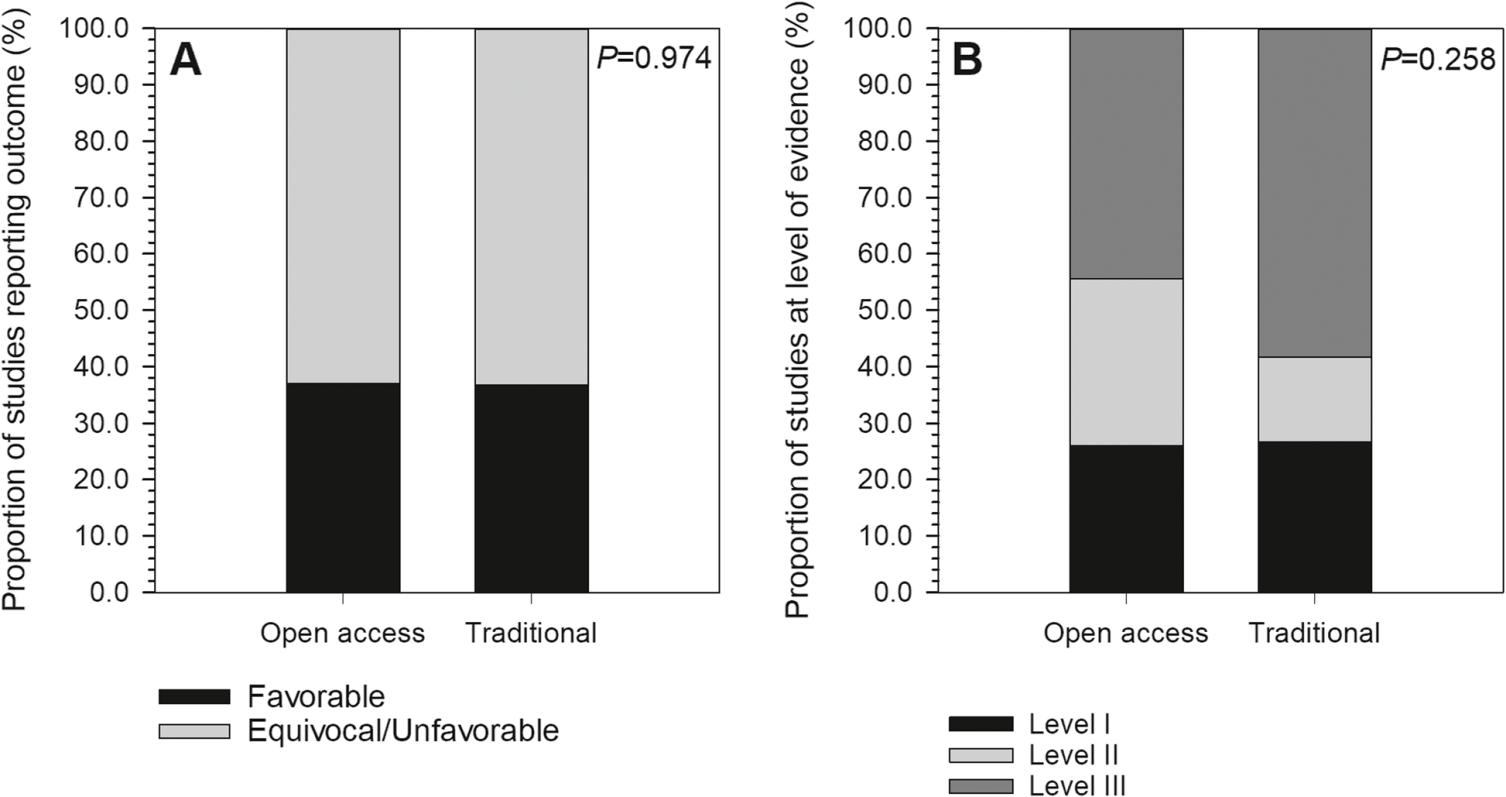
**A** Proportions of studies reporting favorable outcomes (black bar segments) or equivocal/unfavorable outcomes (gray bar segments), with comparison between studies published in open access *vs*. traditional journals. Outcome proportions were not significantly different between journal types. **B** Proportions of studies reporting Level I evidence (black bar segments), Level II evidence (light gray bar segments) or Level III evidence (dark gray bar segments), with comparison between studies published in open access vs. traditional journals. Level of evidence distributions were not significantly different between journal types

A total of 87 studies met the inclusion criteria for analysis (see Appendix 1 for a full listing). Of these, 35 studies (40.2%) included at least one author reporting a COI, and 13 studies (14.9%) disclosing receipt of industry funding (Fig. 2). Among all authors, the median for reported industry payments was $0.00 (IQR: $0.00–$569.27; range: $0.00–$27,330,196.00). Within only those authors reporting payments, mean payment was $2,932,099.20 ± $6,997,227.50 (median, $186,474.33; range, $34.96–$27,330,196.00). Most studies (54.0%) presented Level III evidence, with 19.5% at Level II and 26.4% at Level I (Fig. 3). In terms of journals in which included studies were published, mean IF was 2.27 ± 1.41 (range, 0.14–5.85), mean RCR was 3.38 ± 3.45 (range, 0.00–16.74), and 31.0% were open access (Figs. 4 & 5). Overall study outcomes were 36.8% favorable, 49.4% equivocal, and 13.8% unfavorable.

Conflicts and industry funding had no significant effects on study results (respectively: χ^*2*^ = 0.16, *P* = 0.682; χ^2^ = 0.58, *P* = 0.447), although there was a trend toward studies disclosing industry funding being more likely to report favorable results (Table 1). There were also no significant effects of conflicts (χ^*2*^ = 1.35, *P* = 0.508) or funding (χ^*2*^ = 0.38, *P* = 0.826) on study level of evidence (Table 2). Studies in which any author(s) disclosed a conflict had significantly higher RCR (*S* = 1041, *P* < 0.001) and IF (*S* = 1788, *P* = 0.032) than studies reporting no conflicts, but the same was not true of disclosures of industry funding (RCR: *S* = 349, *P* = 0.737; IF: *S* = 569, *P* = 0.976) (Table 3). Study results were not significantly related to RCR (favorable, 3.3 ± 2.9; equivocal/ unfavorable, 3.4 ± 3.8; *S* = 740.5, *P* = 0.846) or IF (favorable, 2.0 ± 1.5; equivocal/ unfavorable, 2.4 ± 1.4; *S* = 1211.5, *P* = 0.084), nor were they significantly related to level of evidence (χ^*2*^ = 2.79, *P* = 0.245) despite a trend toward favorable studies more frequently relying on Level III evidence (favorable, 65.5% Level III *vs*. equivocal/unfavorable, 47.3% Level III). Publication in an open access journal was related neither to level of evidence (χ^*2*^ = 2.71, *P* = 0.258), nor study results (χ^*2*^ = 001, *P* = 0.974) (Table 4).

**Table 1:** Effects of conflicts of interest and industry funding on study results

Results	Any author(s) reporting conflicts		Disclosure of industry funding		Total industry payments: median (IQR; range)
	Yes	No	Yes	No	
Equivocal/Unfavorable	65.7%	64.5%	53.9%	64.9%	$0.00 ($0.00–$782.07; $0.00–$27,330,196.00)
Favorable	34.3%	38.5%	46.2%	35.1%	$0.00 ($0.00–$26.22; $0.00–$6,810,167.80)
	χ^*2*^ = 0.16, *P* = 0.682		χ^*2*^ = 0.58, *P* = 0.447		*S* = 1377, *P* = 0.737

**Table 2:** Effect of conflicts of interest and industry funding on level of evidence

Level of Evidence	Any author(s) reporting conflicts		Disclosure of industry funding	
	Yes	No	Yes	No
I	20.0%	30.8%	30.8%	25.7%
II	22.9%	17.3%	23.1%	18.9%
III	57.1%	51.9%	46.2%	55.4%
	χ^*2*^ = 1.35, *P* = 0.508		χ^*2*^ = 0.38, *P* = 0.826

**Table 3:** Effect of conflicts of interest and industry funding on RCR and IF

Level of Evidence	Any author(s) reporting conflicts			Disclosure of industry funding		
	Yes	No	*S*, P*	Yes	No	*S*, P*
RCR	5.2 ± 4.4	2.3 ± 2.2	1041, < 0.001	3.0 ± 2.1	3.4 ± 3.7	349, 0.737
IF	2.8 ± 1.6	1.9 ± 1.2	1788, 0.032	2.3 ± 1.4	2.3 ± 1.4	569, 0.976

^*^Statistical results for Wilcoxon-Mann-Whitney tests

**Table 4:** Effect of open access on level of evidence and study results

Outcome Measures	Open Access	
	Yes	No
Level of Evidence		
I	25.9%	26.7%
II	29.6%	15.0%
III	44.4%	58.3%
Study result		
Favorable	37.0%	63.0%
Equivocal/Unfavorable	36.7%	63.3%

A subanalysis by emerging technology subtype was also performed. Both RA and PSI studies more frequently reported COI/industry funding than CA or UKA studies (*P* ≥ 0.045). Technology subtype also significantly influenced study outcomes (*P* = 0.013), particularly in the case of RA and UKA, which were both more likely to report favorable outcomes than PSI studies (RA *vs*. PSI: OR = 6.31, 95% CI: 1.61–24.68; UKA *vs*. PSI: OR = 9.14, 95% CI: 1.43–58.53).

## Discussion

New and innovative technologies are attractive for their potential to improve TJA results. While new technologies such as RA demonstrate the ability to improve radiological outcomes, it remains unclear whether these benefits translate to improved clinical and patient-reported outcomes. Physicians seeking to adopt new technologies into their practice must critically assess the evidence, including evaluating the potential influence of author COI and/or industry funding on study outcomes. The current investigation reviewed the body of literature evaluating non-radiographic, functional and patient-reported outcomes of emerging technologies for hip and knee arthroplasty, finding that neither author COI nor industry funding had a significant effect on study results or study level of evidence. However, studies in which any author reported a COI had a significantly higher RCR and journal IF than non-conflicted studies.

COI can influence how studies are designed, conducted, analyzed, and reported, and both author financial COIs and commercial funding have been demonstrated to be associated with more frequent reporting of statistically significant results and favorable study conclusions [30, 31]. The specific influence of COIs in the orthopedic literature, and even more specifically in the arthroplasty literature, has not been consistent. Early studies on the topic demonstrated strong associations between industry funding and favorable outcomes in articles published in major orthopedic subspecialty journals [32, 33]. Leopold and associates [32] reviewed articles from 3 prominent orthopedic journals (The Journal of Bone and Joint Surgery, The American Journal of Sports Medicine, and The Journal of Arthroplasty) published between 1999–2000 and demonstrated that receipt of commercial funding was the only variable analyzed that was found to be significantly associated with a positive study outcome. Interestingly, the authors found that the apparent association between funding and outcome was stronger in The Journal of Arthroplasty than in either of the other journals reviewed, which they noted may be a function of a substantial industry presence in the subspecialty of joint replacement.

More recent investigations of the influence of COIs on study conclusions in various other orthopedic subspecialty fields have produced conflicting results [28, 34–36]. In the current investigation, studies of emerging TJA technologies in which any author reported a COI were not more likely to report favorable results than those without any author COIs. Furthermore, studies involving industry funding were also not more likely to report favorable results when compared to those without commercial funding. These findings contrast with a recent systematic review of studies evaluating the use of robotics in TKA, THA, and UKA [21].

DeFrance and colleagues reviewed studies comparing robotic-assisted arthroplasty to conventional arthroplasty and found that conflicted studies were more likely to report favorable results of robotics than non-conflicted studies [21]. They also noted that the vast majority (91%) of studies had at least one author with a COI and that most studies (78%) reported favorable conclusions. In the current investigation, a much smaller percentage of studies had an author with a COI (40%) and a much smaller percentage of studies reported favorable results (37%). These discrepancies may be due to differences in the study selection criteria. DeFrance *et al*. included all studies of RA arthroplasty which included a comparison between conventional and RA techniques. A significant percentage of those studies evaluated only radiographic outcomes, and were therefore excluded from the current investigation. Of the 54 studies included in that review, only 6 (11.1%) were included in the current investigation. The results presented by DeFrance *et al*., therefore, likely better reflect the RA arthroplasty literature as a whole, while those of the current study may represent a smaller percentage of the literature.

There is an abundance of studies regarding new arthroplasty technologies, many of which report only radiographic outcome measures (*e*.*g*., accuracy of component positioning). However, the clinical relevance of such outcomes has not been fully elucidated [24–27]. We aimed to determine whether financial COI influenced the conclusions of those studies presenting only what we believed to be the most clinically meaningful outcomes to patients and physicians, in order to provide immediate and practical relevance to better inform shared decision-making when considering the use of new technologies in patient care. While DeFrance *et al*. found that the majority of all RA arthroplasty studies were conflicted and that conflicted studies were more likely to report favorable conclusion, our results suggest that funding and author financial COI are less prevalent and have no influence on the conclusions of the most clinically relevant studies of emerging TJA technologies.

The association between author COIs and study bibliometrics has been scarcely reported. Grundy *et al*. reviewed articles published in medical journals across all fields during the year 2016 and found that conflicted studies were published in journals with higher IF and received more attention in the scientific literature and media (as measured by Altmetric scores) [37]. Similarly, Okike *et al*. reviewed articles published in 3 frequently cited orthopedic journals during 2002–2003 and demonstrated that self-reported author COIs, including both those related to non-profit organizations (*i*.*e*., non-industry) and those related to for-profit companies (*i*.*e*., industry), were associated with higher rates of citation [38]. Finally, DeFrance *et al*. reported no significant difference in journal IF between conflicted and non-conflicted robotic arthroplasty studies [21]. We found author COIs, but not receipt of industry funding, to be associated with higher citation rates and higher journal IFs. The specific explanations for these findings are unclear.

There are also few studies evaluating the association between COIs and study level of evidence in the orthopedic and arthroplasty literature. Narain *et al*. reviewed articles reporting outcomes of cervical disc arthroplasty and found that conflicted studies were significantly more likely to present level I evidence and less likely to present level IV evidence than non-conflicted studies [28]. On the other hand, Mayo *et al*. found that level of evidence was not associated with COIs in a review of studies reporting outcomes of autologous chondrocyte implantation [36]. DeFrance *et al*. also reported no difference in level of evidence between conflicted and non-conflicted studies of robotic hip and knee arthroplasty [21]. Similarly, we found that neither COIs nor industry funding had a significant effect on level of evidence in studies reporting outcomes of emerging TJA technologies.

This study is not without limitations. First, only studies with reported functional outcomes comparing conventional instrumentation to an emerging technology were included. Although a large body of literature focused on radiographic outcomes and laboratory values, these were not the focus of the present study and our results therefore may not be representative of the entire body of literature regarding emerging TJA technologies. These stringent inclusion/exclusion criteria also produced a relatively small sample, which may have resulted in statistical tests being underpowered. Our analysis included “all comers” to emerging technology, and we did not differentiate between specific technologies and industry manufacturer. Open Payments, currently the most accepted comprehensive and regulated database of funding, was utilized in determining the primary outcome measures of our study. Therefore, studies published before 2013 could not be included and it is possible that inaccuracy in data reporting is present. All data regarding COI and funding were obtained directly from the original, full-text publication which inherently relies upon the accuracy with which these were reported and subsequently published. Finally, there is the risk of introducing bias into the analysis given that study outcome grading was subjectively determined from our authors' assessment of study findings.

## Conclusions

The results of the current study suggest that author COIs are prevalent in the published body of literature reporting outcomes of emerging TJA technologies, but the percentage of conflicted studies (about 40%) is lower than that previously reported for other orthopedic subspecialty technologies/techniques. Studies utilizing RA and PSI more frequently report COI, while RA and UKA studies more often report favorable outcomes than PSI. No statistically significant association was found between the presence of author COIs and/or industry funding and the frequency of favorable outcomes or study level of evidence. Physicians should still critically evaluate published articles for COIs and consider the effects of potential bias before implementing new technologies into practice.

## Supplementary Information

**Additional file 1:** **Appendix 1.** Included emerging technology TJA studies.

## Data Availability

The datasets used and/or analyzed during the current study are available from the corresponding author on reasonable request.

## Abbreviations

PSI: Patient-specific implant/instrumentation
CA: Computer-assisted
RA: Robotic-assisted
COI: Conflict of interest
RCR: Relative citation ratio
IF: Impact factor
UKA: Unicompartmental knee arthroplasty
TJA: Total joint arthroplasty
CMS: Centers for Medicare and Medicaid Services
