# Supplementary material for: Funding has no effect on clinical outcomes of total joint arthroplasty emerging technologies: a systematic review of bibliometrics and conflicts of interest
Source: Arthroplasty. 2022 Nov 1;4:45. doi: 10.1186/s42836-022-00146-3 (PMC9623935; doi:10.1186/s42836-022-00146-3)
Supplement: Supplementary file 1 — Additional file 1: Appendix 1. Included emerging technology TJA studies. [file 42836_2022_146_MOESM1_ESM.docx]

**Appendix 1.** Included emerging technology TJA studies

| **Author (et al.)** | **Article Title** | **Journal** | **Year** | **Technology Type^a^** | **Conclusion^b^** | **Level of Evidence** | **Disclosed Conflict** | **Mean Author Payment ($USD)** | **Journal Impact Factor** | **Relative Citation Ratio** | **Open Access** |
| --- | --- | --- | --- | --- | --- | --- | --- | --- | --- | --- | --- |
| Kim | Less Femoral Lift-Off and Better Femoral Alignment in TKA Using Computer-Assisted Surgery | Knee Surg Sports Traumatol Arthrosc | 2012 | CA | Favorable | 4 | N | 0.00 | 4.342 | 1.23 | N |
| Tibesku | Benefits of Using Customized Instrumentation in Total Knee Arthroplasty: Results from an Activity-Based Costing Model | Arch Orthop Trauma Surg | 2012 | PSI | Favorable | 3 | Y | 0.00 | 3.067 | 3.59 | N |
| Allen | Does Computer-Assisted Total Knee Arthroplasty Improve The Overall Component Position and Patient Function? | Int Orthop | 2013 | CA | Against | 1 | N | 0.00 | 3.075 | 1.16 | N |
| Abd Razak | Computer Navigation Results in Less Severe Flexion Contracture Following Total Knee Arthroplasty | J Arthroplasty | 2013 | CA | Favorable | 3 | N | 0.00 | 4.757 | 0.28 | N |
| Blakeney | Functional Outcomes Following Total Knee Arthroplasty: A Randomised Trial Comparing Computer-Assisted Surgery with Conventional Techniques | Knee | 2013 | CA | Favorable | 1 | N | 0.00 | 2.199 | 2.72 | N |
| Huang | Computed Tomography Evaluation in Total Knee Arthroplasty: Computer-Assisted Navigation Versus Conventional Instrumentation In Patients with Advanced Valgus Arthritic Knees | J Arthroplasty | 2013 | CA | Equivocal | 3 | Y | 0.00 | 4.757 | 1.59 | N |
| Lad | Component Alignment and Functional Outcome Following Computer Assisted and Jig Based Total Knee Arthroplasty | Indian J Orthop | 2013 | CAS | Equivocal | 2 | N | 0.00 | 1.251 | 0.81 | Y |
| Lin | Comparison of The Clinical and Radiological Outcomes of Three Minimally Invasive Techniques for Total Knee Replacement at Two Years | Bone Joint J | 2013 | CA | Against | 1 | N | 0.00 | 5.082 | 1.51 | N |
| Liow | Robot-Assisted Total Knee Arthroplasty Accurately Restores the Joint Line and Mechanical Axis. A Prospective Randomised Study | J Arthroplasty | 2013 | RA | Favorable | 1 | N | 0.00 | 4.757 | 6.27 | N |
| Lutzner | No Difference Between Computer-Assisted and Conventional Total Knee Arthroplasty: Five-Year Results of a Prospective Randomised Study | Knee Surg Sports Traumatol Arthrosc | 2013 | CA | Equivocal | 1 | N | 0.00 | 4.342 | 3.12 | N |
| Nakano | Long-Term Subjective Outcomes of Computer-Assisted Total Knee Arthroplasty | Int Orthop | 2013 | CA | Equivocal | 3 | N | 0.00 | 3.075 | 1.06 | N |
| Song | Robotic-Assisted TKA Reduces Postoperative Alignment Outliers and Improves Gap Balance Compared to Conventional TKA | Clin Orthop Relat Res | 2013 | RA | Equivocal | 1 | Y | 0.00 | 4.176 | 8.08 | N |
| Thiengwittayaporn | Midterm Outcomes of Electromagnetic Computer-Assisted Navigation in Minimally Invasive Total Knee Arthroplasty | J Orthop Surg Res | 2013 | CA | Equivocal | 1 | N | 0.00 | 2.359 | 0.97 | Y |
| Yaffe | Computer-Assisted Versus Manual TKA: No Difference in Clinical or Functional Outcomes at 5-Year Follow-Up | Orthopedics | 2013 | CA | Equivocal | 4 | Y | 0.00 | 1.390 | 0.69 | N |
| Yaffe | Clinical, Functional, and Radiographic Outcomes Following Total Knee Arthroplasty with Patient-Specific Instrumentation, Computer-Assisted Surgery, and Manual Instrumentation: A Short-Term Follow-Up Study | Int J Comput Assist Radiol Surg | 2013 | PSI, CA | Favorable | 3 | N | 0.00 | 2.924 | 2.76 | N |
| Zhang | Minimal Invasive and Computer‐Assisted Total Knee Replacement Compared with the Minimal Invasive Technique: A Prospective, Randomized Trial with Short‐Term Outcomes | Arch Orthop Trauma Surg | 2013 | CA | Equivocal | 1 | N | 0.00 | 3.067 | 0.19 | N |
| Zhang | Minimally Invasive and Computer-Assisted Total Knee Arthroplasty Versus Conventional Technique: A Prospective, Randomized Study | Eur J Orthop Surg Traumatol | 2013 | CA | Favorable | 1 | N | 0.00 | 0.181 | 0.55 | N |
| Abdel | No Benefit of Patient-Specific Instrumentation in TKA On Functional and Gait Outcomes: A Randomized Clinical Trial | Clin Orthop Relat Res | 2014 | PSI | Equivocal | 1 | Y | 56.88 | 4.176 | 4.32 | N |
| Blyth | Electromagnetic Navigation in Total Knee Arthroplasty—A Single Center, Randomized, Single-Blind Study Comparing the Results with Conventional Techniques | J Arthroplasty | 2014 | CA | Equivocal | 1 | N | 0.00 | 4.757 | 0.64 | N |
| Brown | Imageless Computer-Assisted Versus Conventional Total Hip Arthroplasty: One Surgeon’s Initial Experience | J Arthroplasty | 2014 | CA | Against | 3 | N | 569.27 | 4.757 | 1.6 | N |
| Chen | Sagittal Component Alignment is Less Reliable Than Coronal Component Alignment in a Chinese Population Undergoing Navigated TKA | J Orthop Surg Res | 2014 | CA | Against | 3 | N | 0.00 | 2.359 | 0.00 | Y |
| Cip | Conventional Versus Computer-Assisted Technique for Total Knee Arthroplasty: A Minimum Of 5-Year Follow-Up of 200 Patients in a Prospective Randomized Comparative Trial | J Arthroplasty | 2014 | CA | Equivocal | 1 | N | 5,000.00 | 4.757 | 3.03 | N |
| DeHaan | Patient-Specific Versus Conventional Instrumentation for Total Knee Arthroplasty: Peri-Operative and Cost Differences | J Arthroplasty | 2014 | PSI | Favorable | 3 | Y | 40,210.27 | 4.757 | 3.12 | N |
| Huang | Comparison of Computer-Navigated and Conventional Total Knee Arthroplasty in Patients with Ranawat Type-II Valgus Deformity: Medium-Term Clinical and Radiological Results | BMC Musculoskelet Disord | 2014 | CA | Equivocal | 3 | N | 0.00 | 2.362 | 0.93 | Y |
| Huang | Differences in Component and Limb Alignment Between Computer-Assisted and Conventional Surgery Total Knee Arthroplasty | Knee Surg Sports Traumatol Arthrosc | 2014 | CA | Equivocal | 3 | N | 0.00 | 4.342 | 1.95 | Y |
| Kamat | Total Knee Replacement in The Obese Patient: Comparing Computer Assisted and Conventional Technique | ScientificWorldJournal | 2014 | CA | Favorable | 3 | N | 0.00 | 1.219 | 0.57 | Y |
| Lee | The Benefits of Computer-Assisted Total Knee Arthroplasty on Coronal Alignment with Marked Femoral Bowing in Asian Patients | J Orthop Surg Res | 2014 | CA | Equivocal | 3 | N | 0.00 | 2.359 | 1.37 | Y |
| Thienpont | The Use of Patient-Specific Instruments Does Not Reduce Blood Loss During Minimally Invasive Total Knee Arthroplasty? | Knee Surg Sports Traumatol Arthrosc | 2014 | PSI | Equivocal | 3 | N | 0.00 | 4.342 | 1.64 | N |
| Woolson | Component Alignment During Total Knee Arthroplasty with Use of Standard or Custom Instrumentation | J Bone Joint Surg Am | 2014 | PSI | Against | 1 | Y | 140.45 | 5.284 | 6.29 | N |
| Yan | Comparison Between Patient‐Specific Instruments and Conventional Instruments and Computer Navigation in Total Knee Arthroplasty: A Randomized Controlled Trial | Knee Surg Sports Traumatol Arthrosc | 2014 | PSI, CA | Against | 1 | N | 0.00 | 4.342 | 4.55 | N |
| Zhang | Superior Alignment but No Difference in Clinical Outcome After Minimally Invasive Computer‐Assisted Unicompartmental Knee Arthroplasty (MICA‐UKA) | Knee Surg Sports Traumatol Arthrosc | 2014 | UKA / CA | Favorable | 2 | N | 0.00 | 4.342 | 1.6 | N |
| Kim | Results of Gender-Specific Total Knee Arthroplasty: Comparative Study with Traditional Implant in Female Patients | Knee Surg Relat Res | 2015 | PSI | Equivocal | 3 | N | 0.00 | 0.000 | 0.61 | Y |
| Kotela | Patient-Specific CT-Based Instrumentation Versus Conventional Instrumentation in Total Knee Arthroplasty: A Prospective Randomized Controlled Study on Clinical Outcomes and In-Hospital Data | Biomed Res Int | 2015 | PSI | Equivocal | 1 | N | 0.00 | 3.411 | 1.73 | Y |
| Abane | Patient-Specific Cutting Guides Were Not Better Than Conventional Instrumentation for Total Knee Arthroplasty | J Bone Joint Surg Am | 2015 | PSI | Equivocal | 1 | Y | 0.00 | 5.284 | 0.00 | N |
| Ollivier | No Functional Benefit After Unicompartmental Knee Arthroplasty Performed with Patient-Specific Instrumentation: A Randomized Trial | Clin Orthop Relat Res | 2015 | UKA / PSI | Against | 1 | N | 0.00 | 4.176 | 4.36 | N |
| Renkawitz | Impingement-Free Range of Movement, Acetabular Component Cover and Early Clinical Results Comparing ‘Femur-First’ Navigation and ‘Conventional’ Minimally Invasive Total Hip Arthroplasty | Bone Joint J | 2015 |  | Equivocal | 1 | N | 0.00 | 5.082 | 4.14 | N |
| Thienpont | The Indirect Cost of Patient-Specific Instruments | Acta Orthop Belg | 2015 | PSI | Against | 3 | N | 0.00 | 0.500 | 1.26 | N |
| White | Patient-Specific Total Knees Demonstrate a Higher Manipulation Rate Compared to “Off-The-Shelf Implants” | J Arthroplasty | 2015 | PSI | Equivocal | 3 | Y | 1,002,621.03 | 4.757 | 2.76 | N |
| Zhu | Outcomes Following Total Knee Arthroplasty with CT‐Based Patient‐Specific Instrumentation | Knee Surg Sports Traumatol Arthrosc | 2015 | PSI | Against | 2 | N | 0.00 | 4.342 | 2.86 | N |
| Bae | A Comparison of the Medium-Term Results of Total Knee Arthroplasty Using Computer-Assisted and Conventional Techniques to Treat Patients with Extraarticular Femoral Deformities | J Arthroplasty | 2016 | CA | Equivocal | 3 | N | 0.00 | 4.757 | 0.86 | N |
| Baumbach | 10-Year Survival of Navigated Versus Conventional TKAs: A Retrospective Study | Orthopedics | 2016 | CA | Favorable | 3 | Y | 0.00 | 1.390 | 2.51 | N |
| Khuangsirikul | 10-Year Patient Satisfaction Compared Between Computer-Assisted Navigation and Conventional Techniques in Minimally Invasive Surgery Total Knee Arthroplasty | Comput Assist Surg (Abingdon) | 2016 | CA | Equivocal | 2 | N | 0.00 | 1.787 | 0.52 | Y |
| Lee | A Comparative Study Between Patient- Specific Instrumentation and Conventional Technique In TKA | Orthopedics | 2016 | PSI | Equivocal | 2 | N | 0.00 | 1.390 | 0.5 | N |
| Liow | Computer-Assisted Stereotaxic Navigation Improves the Accuracy of Mechanical Alignment and Component Positioning in Total Knee Arthroplasty | Arch Orthop Trauma Surg | 2016 | CA | Equivocal | 3 | N | 0.00 | 3.067 | 2.4 | N |
| Nam | The Impact of Custom Cutting Guides on Patient Satisfaction and Residual Symptoms Following Total Knee Arthroplasty | Knee | 2016 | PSI | Against | 3 | Y | 1,702,541.95 | 2.199 | 0.67 | N |
| Song | Comparison of Outcome and Survival After Unicompartmental Knee Arthroplasty Between Navigation and Conventional Techniques with an Average 9-Year Follow-Up | J Arthroplasty | 2016 | UKA / CA | Equivocal | 3 | N | 0.00 | 4.757 | 2.67 | N |
| Alvand | The Impact of Patient‐Specific Instrumentation On Unicompartmental Knee Arthroplasty: A Prospective Randomised Controlled Study | Knee Surg Sports Traumatol Arthrosc | 2017 | UKA / PSI | Against | 1 | N | 0.00 | 4.342 | 3.74 | Y |
| Amundsen | Algorithmic Pie-Crusting of The Medial Collateral Ligament Guided By Sensing Technology Affects the Use of Constrained Inserts During Total Knee Arthroplasty | Int Orthop | 2017 |  | Equivocal | 3 | N | 40,966.29 | 3.075 | 1.2 | N |
| Clement | Articular Surface Mounted Navigated Total Knee Arthroplasty Improves the Reliability of Component Alignment | Knee Surg Sports Traumatol Arthrosc | 2017 | CA | Equivocal | 3 | N | 0.00 | 4.342 | 0.4 | Y |
| Culler | Comparison of Adverse Events Rates and Hospital Cost Between Customized Individually Made Implants and Standard Off-The-Shelf Implants for Total Knee Arthroplasty | Arthroplast Today | 2017 | PSI | Favorable | 3 | Y | 0.00 | 1.610 | 2.63 | N |
| Goh | Accelerometer-Based and Computer-Assisted Navigation in Total Knee Arthroplasty: A Reduction in Mechanical Axis Outliers Does Not Lead to Improvement in Functional Outcomes or Quality of Life When Compared to Conventional Total Knee Arthroplasty | J Arthroplasty | 2017 | CA | Equivocal | 2 | N | 0.00 | 4.757 | 5.89 | N |
| Liow | Robotic‐Assisted Total Knee Arthroplasty May Lead to Improvement In Quality‐Of‐Life Measures: A 2‐Year Follow‐Up of a Prospective Randomized Trial | Knee Surg Sports Traumatol Arthrosc | 2017 | RA | Equivocal | 2 | N | 0.00 | 4.342 | 7.22 | N |
| Marchand | Patient Satisfaction Outcomes After Robotic Arm-Assisted Total Knee Arthroplasty: A Short-Term Evaluation | J Knee Surg | 2017 | RA | Favorable | 3 | N | 1,305,141.01 | 2.757 | 6.63 | N |
| Maus | No Improvement in Reducing Outliers in Coronal Axis Alignment with Patient-Specific Instrumentation | Knee Surg Sports Traumatol Arthrosc | 2017 | PSI | Equivocal | 1 | Y | 0.00 | 4.342 | 3.82 | N |
| Yang | Robotic Total Knee Arthroplasty with a Cruciate-Retaining Implant: A 10-Year Follow-Up Study | Clin Orthop Surg | 2017 | RA | Equivocal | 3 | N | 0.00 | 2.830 | 5.67 | Y |
| Cho | Robotic Versus Conventional Primary Total Knee Arthroplasty: Clinical and Radiological Long-Term Results with A Minimum Follow-Up of Ten Years | Int Orthop | 2018 | RA | Equivocal | 3 | N | 0.00 | 3.075 | 10.57 | N |
| Kayani | Robotic-Arm Assisted Total Knee Arthroplasty is Associated with Improved Early Functional Recovery and Reduced Time to Hospital Discharge Compared with Conventional Jig-Based Total Knee Arthroplasty | Bone Joint J | 2018 | RA | Favorable | 2 | Y | 0.00 | 5.082 | 16.28 | Y |
| Stolarczyk | Does Patient-Specific Instrumentation Improve Femoral and Tibial Component Alignment in Total Knee Arthroplasty? A Prospective Randomized Study | Adv Exp Med Biol | 2018 | PSI | Against | 1 | N | 0.00 | 2.622 | 1.6 | N |
| Stone | Functional Outcomes and Accuracy of Patient-Specific Instruments for Total Knee Arthroplasty | Surg Innov | 2018 | PSI | Equivocal | 3 | Y | 186,474.33 | 2.058 | 1.46 | N |
| Wheatley | Early Outcomes of Patient-Specific Posterior Stabilized Total Knee Arthroplasty Implants | J Orthop | 2018 | PSI | Equivocal | 3 | Y | 139,011.52 | 1.360 | 1.54 | N |
| Attard | Health Costs and Efficiencies of Patient-Specific and Single-Use Instrumentation in Total Knee Arthroplasty: A Randomised Controlled Trial | BMJ Open Qual | 2019 | PSI | Equivocal | 1 | Y | 0.00 | 2.692 | 3.27 | Y |
| Clement | Robot-Assisted Unicompartmental Knee Arthroplasty for Patients with Isolated Medial Compartment Osteoarthritis is Cost-Effective | Bone Joint J | 2019 | UKA / RA | Favorable | 3 | Y | 0.00 | 5.082 | 9.51 | Y |
| Cool | A 90-Day Episode-Of-Care Cost Analysis of Robotic-Arm Assisted Total Knee Arthroplasty | J Comp Eff Res | 2019 | RA | Favorable | 5 | N | 2,722,846.89 | 1.744 | 9.13 | Y |
| Hsu | Comparison of Computer-Assisted Navigation and Conventional Instrumentation for Bilateral Total Knee Arthroplasty | Medicine | 2019 | CA | Equivocal | 1 | N | 0.00 | 1.889 | 2.36 | Y |
| Kayani | An Assessment of Early Functional Rehabilitation and Hospital Discharge in Conventional Versus Robotic-Arm Assisted Unicompartmental Knee Arthroplasty | Bone Joint J | 2019 | UKA / RA | Favorable | 2 | Y | 0.00 | 5.082 | 16.74 | N |
| Khlopas | Patient-Reported Functional and Satisfaction Outcomes After Robotic-Arm-Assisted Total Knee Arthroplasty: Early Results of a Prospective Multicenter Investigation | J Knee Surg | 2019 | RA | Favorable | 2 | Y | 4,748,064.56 | 2.757 | 9.11 | N |
| Koper | No Added Value for Computer-Assisted Surgery to Improve Femoral Component Positioning and Patient Reported Outcomes in Hip Resurfacing Arthroplasty; A Multi-Center Randomized Controlled Trial | BMC Musculoskelet Disord | 2019 | CA | Equivocal | 2 | N | 0.00 | 2.362 | 0.4 | Y |
| León-Munoz | Influence of Instrumentation on the Surgical Time to Implant a Total Knee Prosthesis | Rev Esp Cir Ortop Traumatol | 2019 | CA, PSI | Equivocal | 3 | N | 0.00 | 0.000 | 0.00 | N |
| Marchand | One-Year Patient Outcomes for Robotic-Arm-Assisted Versus Manual Total Knee Arthroplasty | J Knee Surg | 2019 | RA | Favorable | 3 | Y | 1,267,103.91 | 2.757 | 9.48 | N |
| Mont | Health Care Utilization and Payer Cost Analysis of Robotic Arm Assisted Total Knee Arthroplasty at 30, 60, And 90 Days | J Knee Surg | 2019 | RA | Favorable | 3 | Y | 0.00 | 2.757 | 0.00 | N |
| Naziri | Making the Transition from Traditional to Robotic-Arm Assisted TKA: What to Expect? A Single-Surgeon Comparative-Analysis of The First-40 Consecutive Cases | J Orthop | 2019 | RA | Favorable | 3 | Y | 351,999.95 | 1.360 | 6.68 | N |
| O’Connor | The Economic Value of Customized Versus Off-The-Shelf Knee Implants in Medicare Fee-For-Service Beneficiaries | Am Health Drug Benefits | 2019 | PSI | Favorable | 3 | Y | 0.00 | 2.070 | 2.66 | N |
| Park | Robot-Assisted Unicompartmental Knee Arthroplasty Can Reduce Radiologic Outliers Compared to Conventional Techniques | PLoS One | 2019 | UKA / RA | Favorable | 3 | N | 0.00 | 3.240 | 1.65 | Y |
| Reimann | Patient Satisfaction - A Comparison Between Patient-Specific Implants and Conventional Total Knee Arthroplasty | J Orthop | 2019 | PSI | Equivocal | 3 | N | 0.00 | 1.360 | 3.88 | N |
| Selvanayagam | A Prospective Randomized Study Comparing Navigation Versus Conventional Total Knee Arthroplasty | J Orthop Surg (Hong Kong) | 2019 | CA | Equivocal | 1 | N | 0.00 | 1.118 | 1.87 | Y |
| Smith | Improved Patient Satisfaction Following Robotic- Assisted Total Knee Arthroplasty | J Knee Surg | 2019 | RA | Favorable | 2 | Y | 1,125,982.49 | 2.757 | 0.00 | N |
| Turgeon | A Double-Blind Randomized Controlled Trial of Total Knee Replacement Using Patient-Specific Cutting Block Instrumentation Versus Standard Instrumentation | Can J Surg | 2019 | PSI | Equivocal | 1 | Y | 127,603.35 | 2.089 | 1.53 | N |
| Bhimani | Robotic-Assisted Total Knee Arthroplasty Demonstrates Decreased Postoperative Pain and Opioid Usage Compared To Conventional Total Knee Arthroplasty | Bone Jt Open | 2020 | RA | Favorable | 3 | Y | 1,185,811.71 | 4.306 | 0.00 | Y |
| Clement | Robotic-Assisted Unicompartmental Knee Arthroplasty Has a Greater Early Functional Outcome When Compared to Manual Total Knee Arthroplasty for Isolated Medial Compartment Arthritis | Bone Joint Res | 2020 | UKA / RA | Favorable | 2 | Y | 0.00 | 5.853 | 5.04 | Y |
| Cotter | Comparative Cost Analysis of Robotic-Assisted and Jig-Based Manual Primary Total Knee Arthroplasty | J Knee Surg | 2020 | RA | Favorable | 3 | Y | 1,509,533.93 | 2.757 | 0.00 | N |
| Grosso | Short-Term Outcomes Are Comparable Between Robotic-Arm Assisted and Traditional Total Knee Arthroplasty | J Knee Surg | 2020 | RA | Equivocal | 3 | Y | 0.00 | 2.757 | 0.00 | N |
| Marshall | Sensor-Guided Knee Surgery Provides Improved Patient Outcomes and Cost Savings in a 90-Day Bundle | Surg Technol Int | 2020 | CA | Favorable | 3 | N | 34.96 | 0.879 | 0.00 | N |
| Mahoney | Improved Component Placement Accuracy with Robotic-Arm Assisted Total Knee Arthroplasty | J Knee Surg | 2020 | RA | Favorable | 2 | Y | 5,466,039.17 | 2.757 | 0.00 | N |
| Nherera | Early Economic Evaluation Demonstrates That Noncomputerized Tomography Robotic-Assisted Surgery is Cost-Effective In Patients Undergoing Unicompartmental Knee Arthroplasty at High-Volume Orthopaedic Centres | Adv Orthop | 2020 | UKA / RA | Equivocal | 5 | N | 0.00 | 0.829 | 0.00 | Y |
| Pelkowski | Robotic-Assisted Versus Manual Total Knee Arthroplasty in a Crossover Cohort: What Did Patients Prefer? | Surg Technol Int | 2020 | RA | Equivocal | 3 | N | 260.69 | 0.879 | 0.00 | N |
| Pierce | Robotic Arm–Assisted Knee Surgery: An Economic Analysis | Am J Manag Care | 2020 | RA | Favorable | 3 | Y | 1,605,678.84 | 2.229 | 1.99 | N |
| Xing | A Comparative Study of Patients’ Subjective Feelings Toward Total Hip Arthroplasty with Patient- Specific Instruments and Traditional Total Hip Arthroplasty | Orthop Surg | 2020 | PSI | Equivocal | 2 | N | 0.00 | 2.071 | 0.00 | Y |
| Yu | Alignment Results of Infrared Computer-Assisted Navigation of Total Knee Arthroplasty for End-Stage Knee Osteoarthritis | Am J Transl Res | 2020 | CA | Favorable | 3 | N | 0.00 | 4.060 | 0.00 | N |
| Clement | Robotic Arm-Assisted Versus Manual Total Hip Arthroplasty | Bone Joint Res | 2021 | RA | Equivocal | 2 | Y | 0.00 | 5.853 | 0.00 | Y |
| Kamalapathy | Navigation Assisted Total Knee Arthroplasty In 54,114 Patients: No Increased Risk in Acute Complications and Hospital Utilisation | Int J Med Robot | 2021 | CA | Favorable | 3 | N | 0.00 | 2.547 | 0.00 | Y |
| Moorthy | Mid‐Term Functional Outcomes of Patient‐Specific Versus Conventional Instrumentation Total Knee Arthroplasty: A Prospective Study | Arch Orthop Trauma Surg | 2021 | PSI | Against | 2 | N | 0.00 | 3.067 | 0.00 | N |
| Negrín | Robotic-Assisted Vs Conventional Surgery in Medial Unicompartmental Knee Arthroplasty: A Clinical and Radiological Study | Knee Surg Relat Res | 2021 | UKA / RA | Favorable | 2 | N | 0.00 | 0.000 | 0.00 | Y |
| Shah | Robotic Total Knee Arthroplasty: A Missed Opportunity for Cost Savings In Bundled Payment for Care Improvement Initiatives? | Surgery | 2021 | RA | Favorable | 3 | N | 0.00 | 3.982 | 0.00 | N |
| Shaw | Minimal Clinically Important Difference in Robotic-Assisted Total Knee Arthroplasty Versus Standard Manual Total Knee Arthroplasty | J Arthroplasty | 2021 | RA | Equivocal | 2 | Y | 481.59 | 4.757 | 0.00 | N |
| Singh | Does the Use of Intraoperative Technology Yield Superior Patient Outcomes Following Total Knee Arthroplasty? | J Arthroplasty | 2021 | CA, RA | Equivocal | 3 | Y | 252,345.97 | 4.757 | 0.00 | N |
| Singh | Robotics Versus Navigation Versus Conventional Total Hip Arthroplasty: Does the Use of Technology Yield Superior Outcomes? | J Arthroplasty | 2021 | CA, RA | Equivocal | 3 | Y | 90,973.43 | 4.757 | 0.00 | N |
| Wang | Patient-Specific Total Hip Arthroplasty is Superior to Conventional Methods for Crowe III and IV Adult Developmental Hip Dysplasia: A Randomized Controlled Trial | Ann Transl Med | 2021 | PSI | Equivocal | 1 | N | 0.00 | 3.932 | 0.00 | Y |
| Zhang | Adoption of Robotic-Arm-Assisted Total Knee Arthroplasty is Associated with Decreased Use of Articular Constraint and Manipulation Under Anesthesia Compared to a Manual Approach | J Knee Surg | 2021 | RA | Favorable | 3 | N | 568,066.62 | 2.757 | 0.00 | N |

^a^Technology types reported as unicompartmental knee arthroplasty (UKA), robotic-assisted (RA), computer-assisted (CA), patient-specific implant (PSI)

^b^Outcome conclusion reported as emerging technology vs. conventional.
